# Phlegmasia Cerulea Dolens, a Deadly Complication of Deep Vein Thrombosis: Case Report and Review of Literature

**DOI:** 10.7759/cureus.19927

**Published:** 2021-11-26

**Authors:** Ban Ibrahim, Raviprasad Kattimani

**Affiliations:** 1 Emergency Medicine, Sunderland and South Tyneside NHS Trust, Newcastle Upon Tyne, GBR; 2 Trauma and Orthopaedics, East Cheshire NHS Trust, Macclesfield, GBR

**Keywords:** phlegmasia cerulea dolens, venous thromboembolism, lung abscess, multiple organ failure, death

## Abstract

Phlegmasia cerulea dolens (PCD) is a rare limb and life-threatening condition caused by extensive deep vein thrombosis of the extremities, and it is classically associated with extensive oedema, severe pain, and skin mottling, which may lead to compartment syndrome, venous gangrene, and even death.

A 40-year-old male, with a background history of right femoral vein thrombosis, cardiomyopathy, with an ejection fraction of only 10%, presented with three days history of progressive swelling, pain, and discolouration of the right lower limb. He was treated with therapeutic low molecular weight heparin along with supportive care with intravenous fluids and pain relief. In view of his poor ejection fraction, he was treated non-operatively with supportive care. The patient succumbed after three months of presentation. PCD is a rare vascular emergency condition that if not recognized early and treated aggressively may lead to higher morbidity and mortality.

## Introduction

Phlegmasia cerulea dolens (PCD) is a rare limb and life-threatening sequela of deep vein thrombosis characterized by pain, swelling, and skin mottling of the extremities [1]. It is commonly associated with hypercoagulable states like malignancy, post-surgery trauma, obesity, and previous thromboembolic conditions. It is more common in males than females [2]. Lower limbs are more frequently involved than upper limbs [2]. Venous thrombosis can progress to dry venous gangrene, sepsis, multi-organ failure and finally death with a high mortality rate [2].

## Case presentation

**Figure 1:**
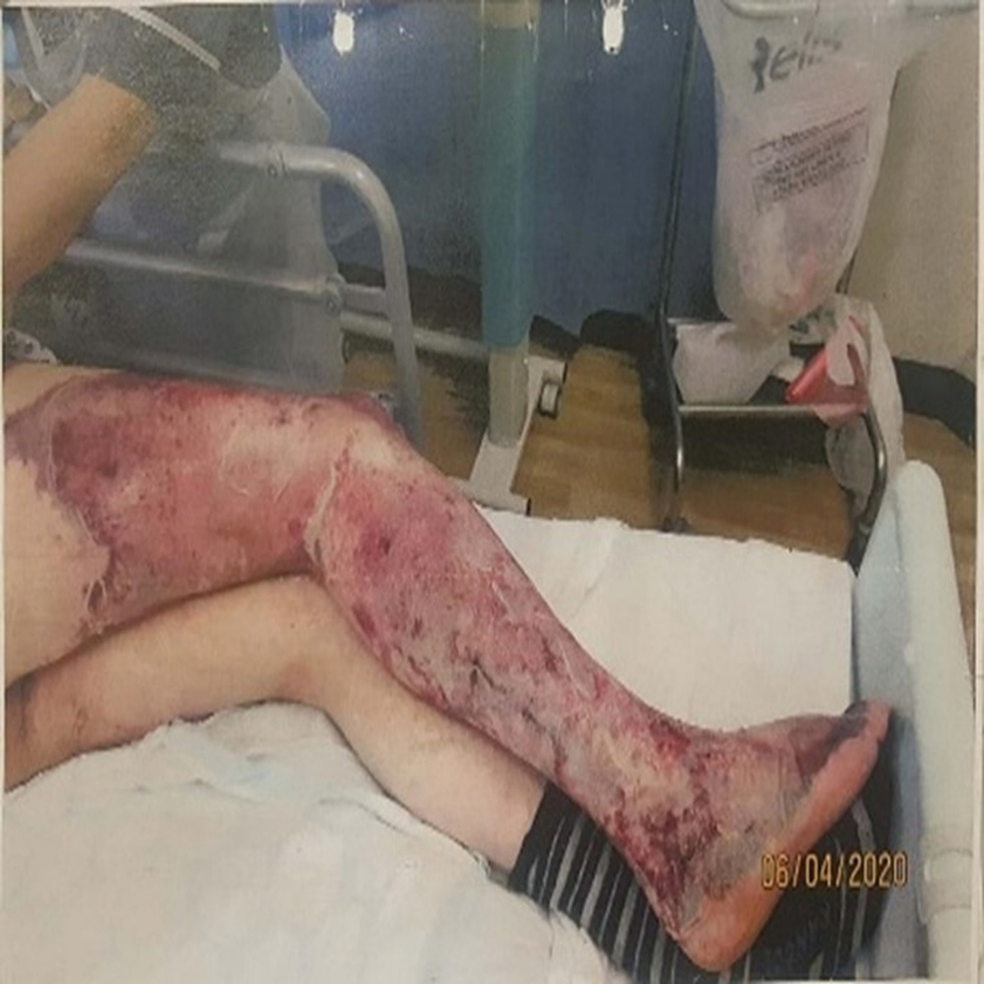
Right lower limb on admission showing lower limb oedema and skin necrosis

**Figure 2:**
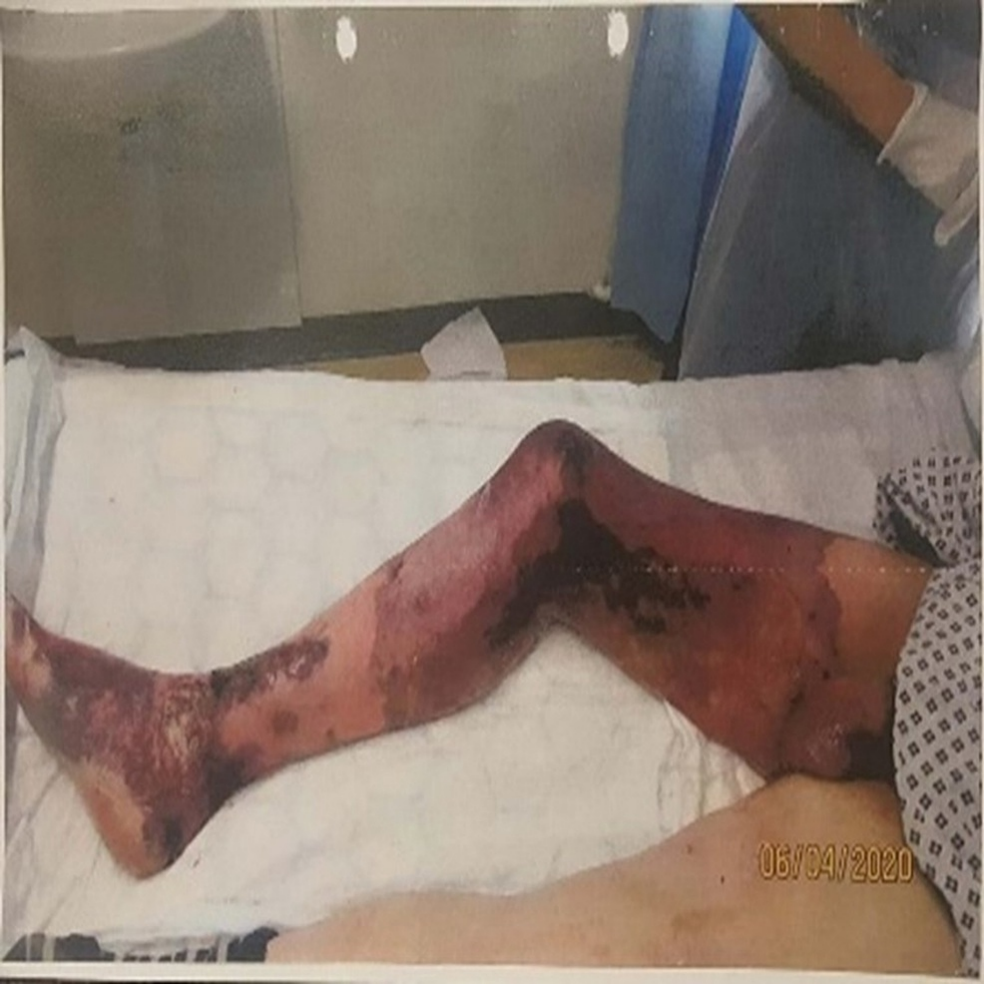
Right lower limb on admission showing lower limb oedema and skin necrosis

A 40-year-old man presented to the accident and emergency department at a district general hospital with three days history of progressive right thigh and leg pain associated with swelling and skin discolouration (Figures 1-2).

He had a background medical history of cardiomyopathy, hypertension, asthma and right femoral vein thrombosis diagnosed five years ago. On examination, he was frail and in septic shock with a blood pressure of 90/60 mmHg and a temperature of 38 degrees Celsius. On local examination of the right lower limb, it had oedema and diffuse bluish discolouration of skin extending from the groin up to the toes. The right lower limb pulses were not palpable. On the day of admission, the blood results showed a white cell count (WCC) of 10.8, C-reactive protein (CRP) of 167, domain dimer (D-dimer) of 7988. He was adequately resuscitated with intravenous fluids and started on broad-spectrum intravenous antibiotics as per the local trust sepsis protocol for three weeks.

**Figure 3:**
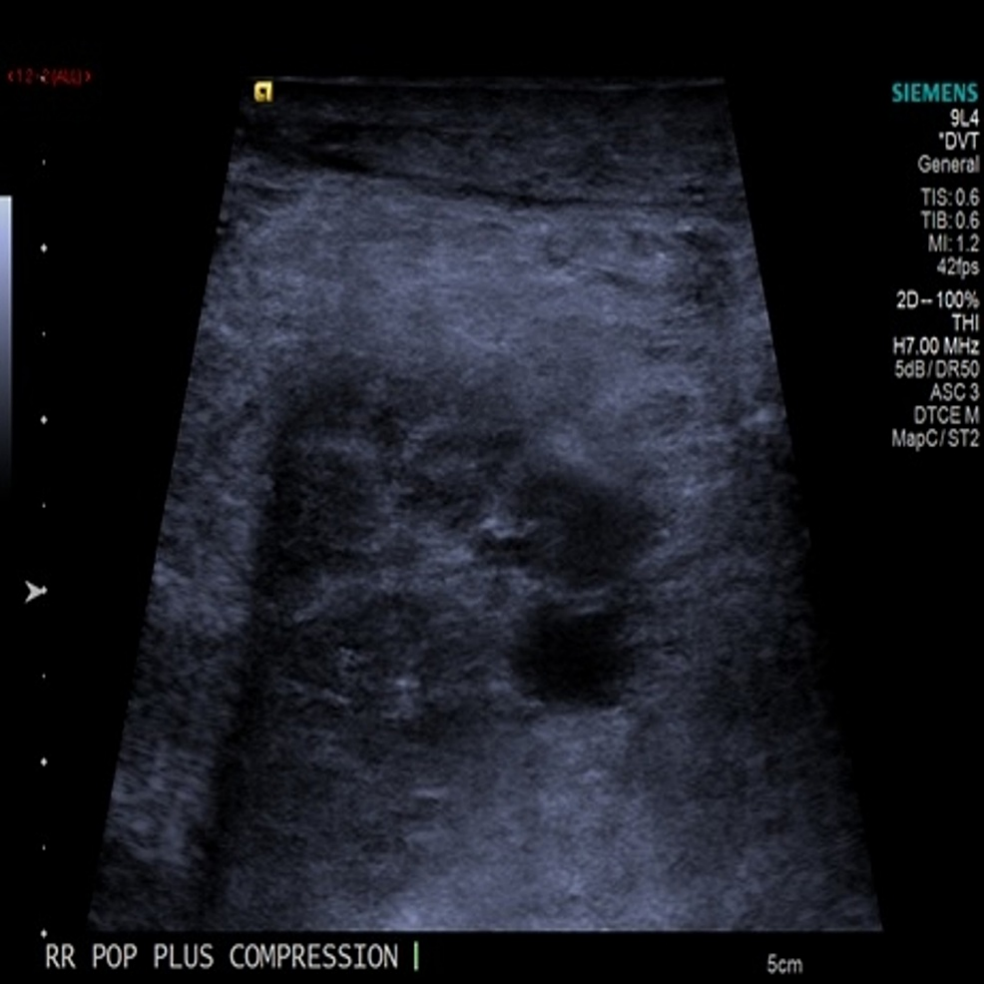
Venous Doppler sonography of the right lower limb; the right popliteal vein is distended with echo poor thrombus

His chest and abdomen radiographs on admission did not show any abnormality. Acute deep vein thrombosis (DVT) involving the right popliteal vein was diagnosed after having venous Doppler sonography the next day of his admission (Figure 3).

**Figure 4:**
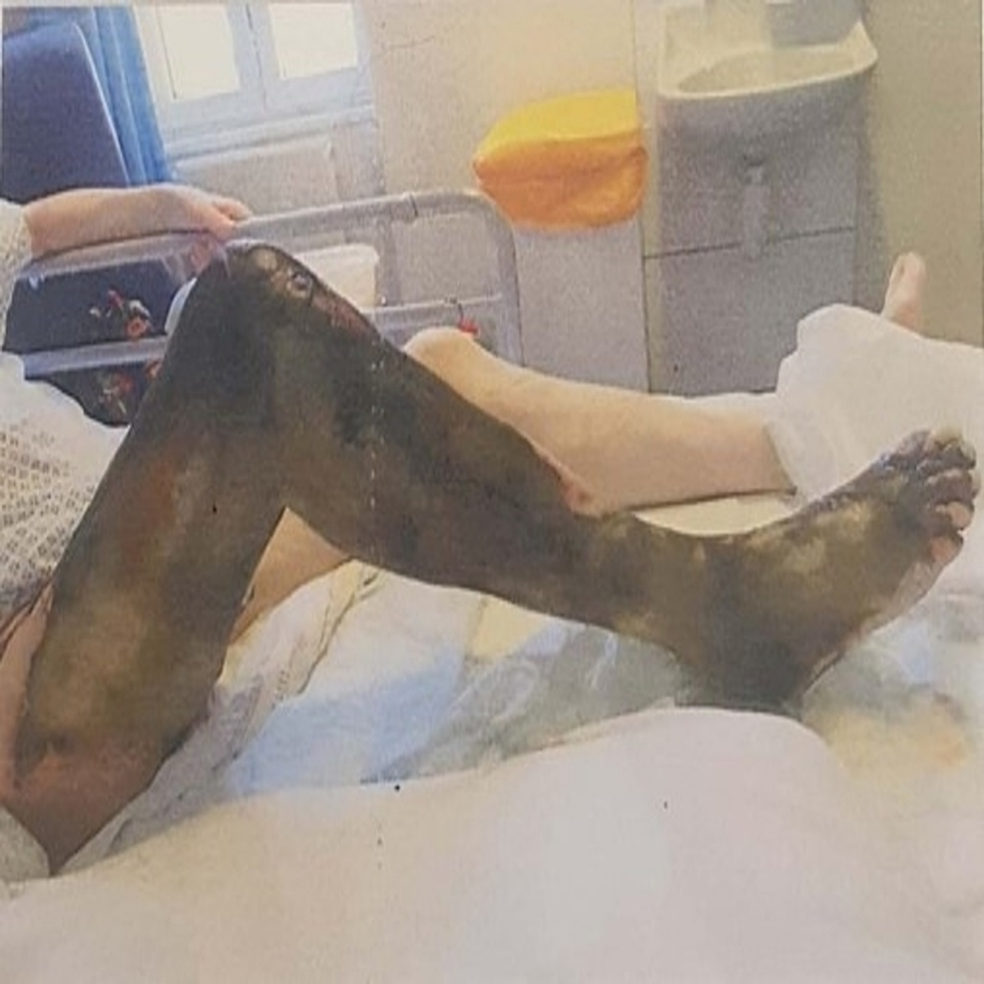
Venous dry gangrene affecting the right lower limb, extending from the toes up to the scrotum after one week of admission

**Figure 5:**
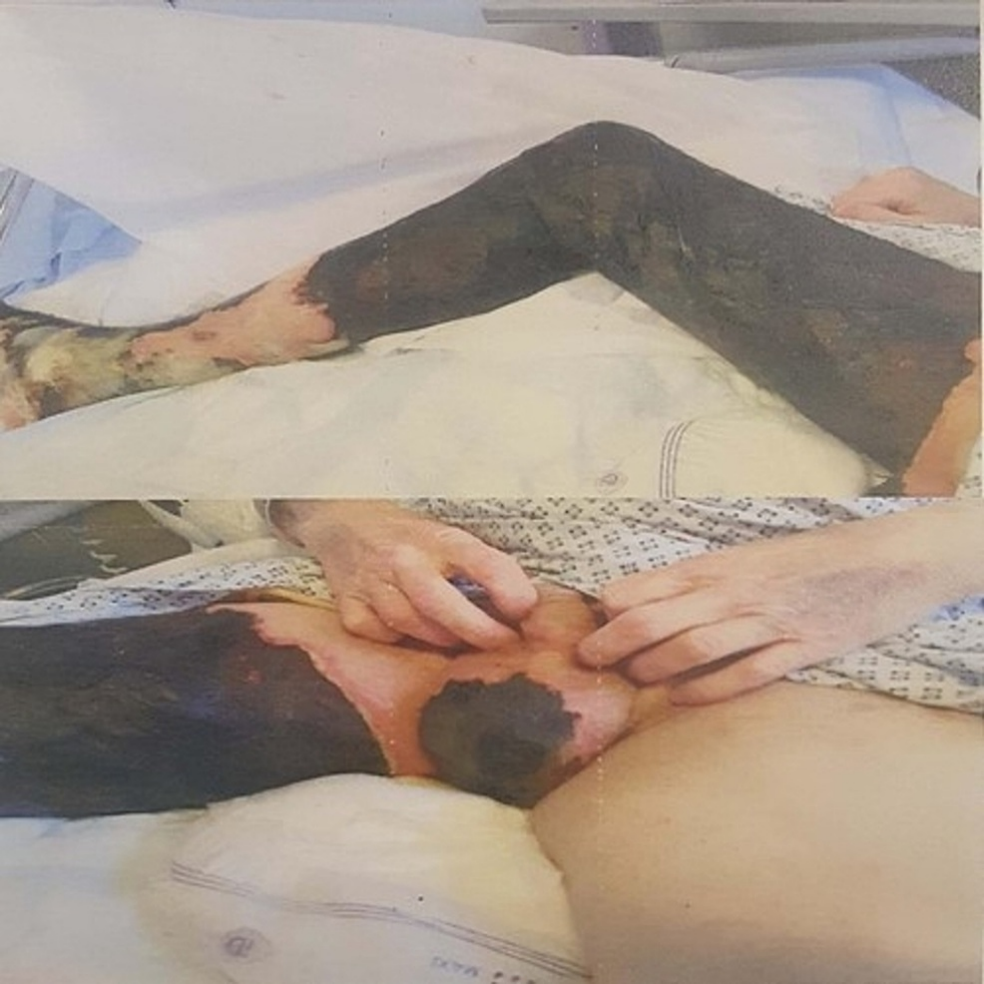
Venous dry gangrene affecting the right lower limb, extending from the toes up to the scrotum after one week of admission

Anti-coagulation with a therapeutic dose of low molecular weight heparin (LMWH) was commenced. Pain relief and limb elevation along with intravenous fluids were given for supportive care. Over a period of two weeks in the hospital, he developed dry gangrene of his leg, extending from the groin up to the toes (Figures 4-5).

**Figure 6:**
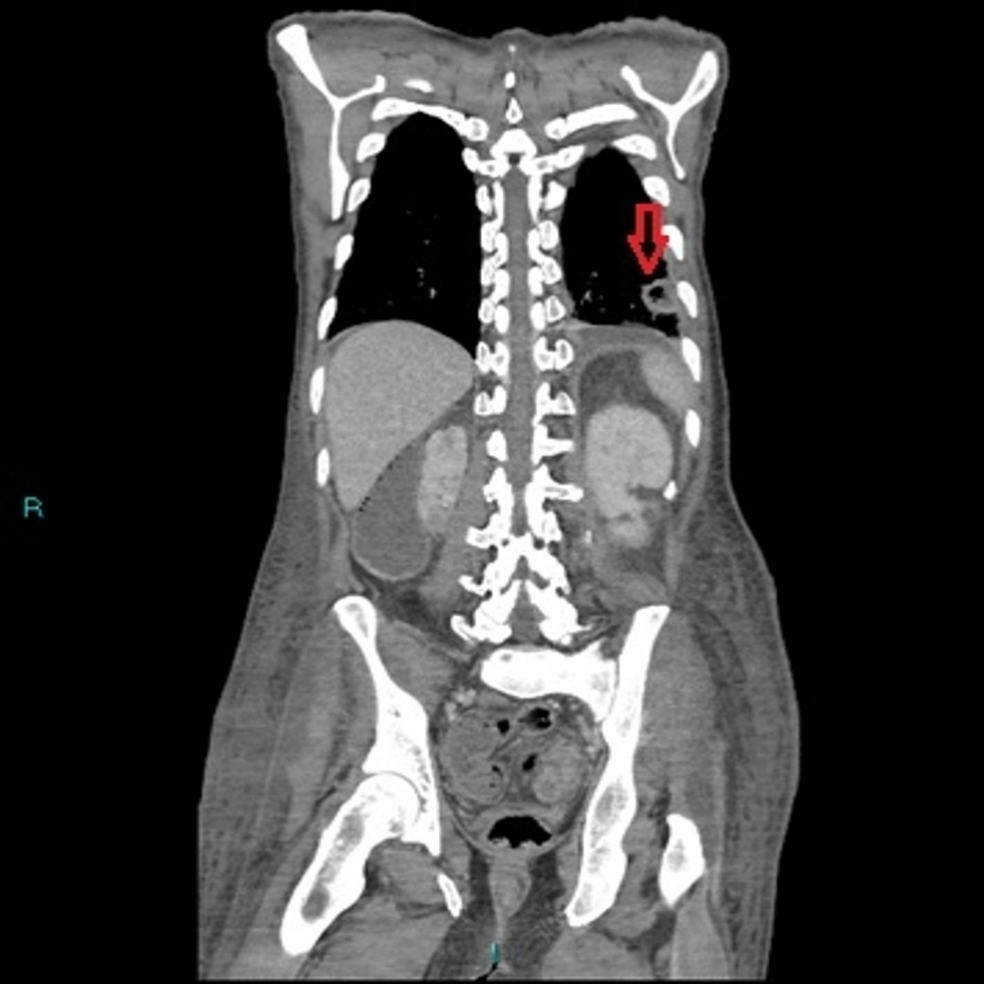
CT thorax, abdomen, and pelvis (TAP) showing a 2.5 cm, sub-pleural, wedge-shaped lesion over the lateral basal segment of the left lower lobe with adjacent atelectasis and small pleural effusion suggestive of abscess

In spite of two weeks of intravenous antibiotics as his inflammatory marker was still raised, with a CRP of 156, to detect the source of infection, he had computed tomography (CT) thorax, abdomen, and pelvis, which revealed a left lower lobe lung abscess (Figure 6).

Transthoracic echocardiography showed a worsening of ejection fraction from 10% on admission to 7%, with severe left ventricular dilatation and severe global hypokinesis of the left ventricle. The outcome of the multi-disciplinary team meeting with plastic and vascular surgeons was to treat the patient non-operatively as he was deemed not fit for surgical intervention for PCD. After three weeks of admission, the patient was discharged to the care of the community hospital to continue anticoagulation and antibiotics along with supportive care for pain relief. Antibiotic treatment was stopped after six weeks, and two weeks following that, his LMWH was converted into a novel oral anti-coagulant. The patient succumbed after 12 weeks of admission.

## Discussion

PCD is a poorly understood condition that results from acute massive venous thromboembolism. The majority of cases involve the lower extremities, with the left limb more commonly involved than the right due to the anatomical relationship between the right iliac artery overlying the left iliac vein; however, it is not uncommon for both lower extremities to be involved [3]. Cancer is the most common aetiology; advanced age, hypercoagulable states, contraceptives, trauma, venous stasis and immobilisation are other contributing factors [4-5]. Venous outflow obstruction causes venous hypertension that leads to increasing interstitial oedema and massive fluid sequestration into the limb. The third space fluid loss leads to hypovolemic shock. The increase in interstitial and compartment pressure ultimately leads to the collapse of the arterial system once the compartment pressure overcomes arterial wall tension, which leads to acute ischemia and venous gangrene increasing patient morbidity and mortality [3]. The diagnosis is mainly based on clinical examination, which shows cardinal signs of skin oedema, pain out of proportion and discolouration or skin mottling [6]. As PCD involves thrombosis of iliac veins, venous ultrasound, which is the first modality of choice to diagnose deep vein thrombosis may not be helpful due to body habitus, depth, overlying bowel gas and incompressibility of the retroperitoneal veins. Computed tomography and magnetic resonance venography can be utilised as an adjunctive imaging modality to detect the extent of proximal thrombus within inferior vena cava and pelvic veins [7]. The sequelae of this disease include venous gangrene in 40%-60%, pulmonary embolism in 50% and amputation in 10%-25% [8]. If symptoms progress to venous gangrene, the risk of amputation is between 20% and 50% with a high rate of mortality from 20% to 40% [9]. Despite the advances in medical science due to the rarity of this condition, there is still no consensus regarding the ideal treatment of PCD [2]. Early diagnosis and supportive treatment with leg elevation, intravenous fluid resuscitation, anticoagulation, along with surgical interventions such as catheter-directed thrombolysis (CDT) or percutaneous thrombectomy, may prevent the complications, as progression to venous gangrene may lead to mortality in up to 57% of cases [10-11]. As per our knowledge, till now there is no case report of PCD associated with a lung abscess and presenting with sepsis. Following the outcome of the discussion of the multi-disciplinary team meeting between the radiological and vascular teams, our patient was deemed not fit for any surgical intervention, including under local anaesthesia due to medical comorbidities and uncertainty of the type of thrombus - whether acute or chronic. Subsequently, his PCD progressed to venous gangrene, which rapidly led to sepsis causing multiple organ failure and death. This case report emphasises the grave outcome of PCD and the importance of early surgical intervention.

## Conclusions

PCD is a life- and limb-threatening condition that must be diagnosed appropriately, and if not treated aggressively with anticoagulation and vascular intervention, there is a higher chance of morbidity with amputation of the limb and mortality.
